# Coronavirus disease 2019 (COVID-19): What we need to know

**DOI:** 10.22088/cjim.11.2.23

**Published:** 2020

**Authors:** Mostafa Javanian, Jila Masrour-Roudsari, Masomeh Bayani, Soheil Ebrahimpour

**Affiliations:** 1 Infectious Diseases and Tropical Medicine Research Center, Health Research Institute, Babol University of Medical Sciences, Babol, Iran

**Keywords:** Coronavirus, COVID-19, Virus, Infection

**Dear Sir,**

In recent years the world has witnessed widespread epidemics of infections as Ebola, Zika and Severe acute respiratory syndrome (SARS). As the repeated alerts have been issued by health organizations, the World Health Organization (WHO) has declared a state of emergency six times in recent decades. It declared 2009 flu pandemic or swine flu, 2014 outbreak of Ebola virus in West Africa, 2014 Polio outbreak in the Middle East, 2016 Zika epidemic in Latin America and the Caribbean, 2018 new case of Ebola in the eastern city of Goma, and 2020 novel Coronavirus in China. 

The identified new coronavirus, called coronavirus disease 2019 (COVID-19) has been first emerged in Wuhan, China, in December 2019, and has now reached several other countries: Hong Kong, Italy, Japan, Russia, Iran, United States, and more than twenty other countries. There are currently 157,215 confirmed cases and 5,843 deaths from COVID-19 outbreak as of March 15, 2020.

Coronaviruses as an RNA viruses are common in several species of animals. Rarely, these viruses may infect humans and then spread among them. It has been confirmed that coronaviruses have frequently made the jump from circulating among animals to presenting new strains to human. The seven coronaviruses that can infect humans, including NL63, HKU1, OC43, 229E, SARS, Middle East Respiratory Syndrome (MERS), and COVID-19 (1). Alongside rhinoviruses, human coronaviruses are responsible for the common cold and mild illness in humans worldwide. The incubation period of this virus remains unidentified. Most evidence declared that the incubation period could be about 5 day and 4–5 days between symptom onset and case detection (2).

The infection can be completely asymptomatic, very severe and even fatal. Common symptoms can include: fever, cough, and shortness of breath. Like many other respiratory infections, the virus is transmitted through droplets from coughs and sneezes. Fecal-oral transmission has also been confirmed for this type of infection. The exact origin of this novel coronavirus remains unknown. Although, some documents have declared like MERS and SARS, all of which have their origins in bats. Some of the cases in China's coronavirus outbreak had linked to a seafood and live animal market, suggesting animal-to-person spread. Even though, some patients did not report any relationship with the animal market, indicating person-to-person spread. The contagious of this virus is less susceptible to other infections like smallpox, measles, polio, acquired immunodeficiency syndrome (AIDS), SARS, and influenza. On average, every person with COVID-19 infects 1.4 to 2.5 people (3). According to reports so far, a fatality rate of COVID-19 is 2% (one case every 50 cases) that compared to SARS mortality (10%) and MERS (over30%) was lower (4). Unfortunately, no antivirals are approved for the treatment of neither COVID-19 infection nor vaccines available for prevention. Therefore, the most important way to prevent infection is to avoid being exposed to this virus like hand and respiratory hygiene.

As we have seen, the control of such infections requires a global consensus given the high rate of their spread, which is easily transferable from one country to another. Patients' information should be made available to health organizations for making an important decision. On the other hand, controlling the spread of emerging and re-emerging viruses like COVID-19 needs serious international collaborations. Moreover, the serious knowledge gaps of the origin, epidemiology, transmission, and other aspects of illness should be complemented by more extensive studies. It is important to note, animal diseases have always been transmitted to humans and in fact most of the new infectious diseases are caused by animals. But environmental changes have accelerated the trend and increased urban populations and international travel have made these diseases grow more rapidly. Therefore, climate change and globalization are changing the way humans and animals are treated, the danger is likely to be greater in the future, and countries need to be on the alert and strengthen their public-health surveillance.
